# The comparison of corneal densitometry in cases with glaucoma following childhood cataract surgery and juvenile glaucoma

**DOI:** 10.1007/s10792-024-03004-0

**Published:** 2024-02-12

**Authors:** Ufuk Elgin, Mert Simsek, Emine Sen, Gozde Hondur, Serdar Bayraktar, Atakan Acar

**Affiliations:** Ulucanlar Eye Education and Research Hospital, University of Health Sciences, Ulucanlar Caddesi No:59, 06240 Altindag Ankara, Turkey

**Keywords:** Aphakic glaucoma, Childhood cataract surgery, Corneal densitometry, Glaucoma, Juvenile glaucoma

## Abstract

**Purpose:**

To compare the corneal densitometry (CD) in pediatric cases with glaucoma following childhood cataract surgery and juvenile open-angle glaucoma (JOAG).

**Methods:**

This prospective comparative study involved 13 eyes with JOAG, 12 eyes with pseudophakic glaucoma, 13 eyes with aphakic glaucoma, and 15 control subjects. Pentacam HR Scheimpflug corneal topography was employed to evaluate corneal thickness (CCT) and CD values.

**Results:**

The mean intraocular pressure (IOP) and CCT were significantly higher in aphakic glaucoma cases than the other groups (*p* = 0.001). In aphakic eyes, the mean CD values were higher in most of the anterior, center, and posterior layers of 0–2 mm, 2–6 mm, 6–10 mm, and total zones (*p* < 0.001 for all). In pseudophakic eyes, the mean CD values were statistically similar with that of aphakic eyes and higher than that of JOAG and control eyes in all layers of 0–2 mm zone and in anterior layer of 10–12 mm and anterior and total layers of 2–6 mm zones (*p* < 0.05 for all). The CD values demonstrated significant correlations with CCT values in both aphakic and pseudophakic eyes. However, a significant correlation of CD values with IOP was only demonstrated in aphakic eyes (*p* = 0.01 for all).

**Conclusion:**

The probable effects of childhood cataract surgery especially aphakia might cause corneal backscatter of light and increased CD in all layers in all zones of the cornea. Increased CD values and its correlation with CCT and IOP in aphakic glaucoma eyes may be of importance in clinical management.

## Introduction

Primary or secondary pediatric glaucoma can lead to childhood blindness. Early diagnosis and treatment are mandatory to prevent structural and functional loss [1–3]. Juvenile open-angle glaucoma (JOAG) is one of the types of primary childhood glaucoma and the prevalence and inheritance patterns are variable in different populations [4–6]. It is more aggressive than primary open-angle glaucoma (POAG) and is known to be associated with higher intraocular pressure (IOP) levels and fluctuations [7].

Glaucoma is one of the most important lifelong complications of pediatric cataract surgery and its incidence is reported to be 15–45% [8, 9]. Almost in one third of the aphakic and pseudophakic glaucoma cases, glaucoma is diagnosed within the first year after cataract surgery, but it may occur even after years or decades [8, 9]. Corneal densitometry (CD) is positively related with corneal backscatter of light but inversely related with corneal transparency [10–12]. The Pentacam Scheimpflug system (Oculus, Wetzlar) can analyze the corneal backscattered light and measure the CD in three layers and four annular zones [10–12].

Here, in this study, the purpose was to compare the CD, possible backscattered light, in pediatric cases with glaucoma following childhood cataract surgery, JOAG and control subjects using Pentacam Scheimpflug corneal tomography. The correlations of the CD values with clinical findings of the study groups were also evaluated.

## Material and methods

This prospective comparative study involved 13 eyes of 13 JOAG cases, 12 eyes of 12 pseudophakic glaucoma cases, 13 eyes of 13 aphakic glaucoma cases, and 15 eyes of 15 control subjects. Glaucoma cases had been under control in glaucoma department and were examined in their follow-up visits. Control subjects had been referred between August 2020 and June 2021. All the study procedures were conducted in accordance with the Declaration of Helsinki, and informed consents were taken from the parents of the participants. This study was approved by the Institutional Review Board and Ethics Committee. All patients were Caucasians.

All the eyes underwent detailed ophthalmological examination. Best-corrected visual acuity (BCVA) with Snellen charts, anterior and posterior segment examination, Goldmann tonometry, or Tonopen (Reichert technology) were performed. Also, gonioscopy with Goldmann three-mirror lens, retinal nerve fiber layer (RNFL), and optic nerve head (ONH) analysis with spectral-domain optical coherence tomography (OCT) (Spectralis Heidelberg Engineering) and visual field analysis (Humphrey Visual Field Analyzer, Carl Zeiss Meditec Inc., Dublin, CA) were performed in cooperative cases before CD measurements. For patients who were not cooperative enough for gonioscopy, the Scheimpflug imaging system (Pentacam, Oculus, Lynwood, WA) was used to screen iridocorneal angle.

All the patients and control subjects were ≤ 16 years-old and glaucoma cases had been under control for at least 6 months. All glaucoma cases had mild-moderate medically controlled glaucoma and none of them had history of any glaucoma surgery. The diagnosis of JOAG based on the optic nerve head changes such as cup-to-disk (C/D) ratio ≥ 0.3 and localized neuroretinal rim defects, IOP higher than 22 mmHg, retinal nerve fiber layer thinning on OCT outside the 95% confidence interval of the normal distribution, and a glaucomatous visual field defect or a glaucoma hemifield test result outside normal limits in cooperative patients. Diagnosis criteria for aphakic and pseudophakic glaucoma were history of cataract surgery for infantile or juvenile cataract and aphakia or pseudophakia in addition to all inclusion criteria for JOAG. All pseudophakic cases had primary intraocular lens (IOL) implantation during cataract surgery. Control subjects had not any ocular problem other than refractive error.

The exclusion criteria were the cases > 16 years-old, primary congenital glaucoma, infantile glaucoma, any other types of secondary glaucoma like iridocorneal endothelial syndrome, Axenfeld–Rieger syndrome, or Peters anomaly, microcornea, nystagmus, any corneal diseases like keratoconus, corneal opacity or any ocular surface diseases, history of previous ocular surgery except cataract surgery for aphakic and pseudophakic eyes, any history of uveitis, trauma, or other inflammatory diseases and contact lens use. Severe glaucoma cases according to Hodapp–Parrish–Anderson classification system in perimetry-cooperated cases and/or history of previous glaucoma surgery were excluded. Also, cases with histories of secondary IOL implantation and juvenile diabetes mellitus were excluded.

The densitometry software of Pentacam HR Scheimpflug corneal topography was used to measure central corneal thickness (CCT) and CD values between 9 am and 12 pm by the same experienced physician under standard dim-light conditions. We evaluated corneal densitometry values of anterior layer (anterior 120 µm), central (between anterior layer and posterior 60 µm), and posterior layer (posterior 60 µm) of 4 annular concentric zones (0–2 mm zone, 2–6 mm zone, 6–10 mm zone, and 10–12 mm zone). The CD values were indicated in grayscale units (GSC) at a range from 0 (maximum transparency) to 100 (total corneal opacity).

SPSS v.21.0 for Windows (SPSS, Inc., Chicago, IL, USA) was employed for statistical analyzes. A prospective power analysis suggested the sample size of at least 11 eyes in each group was enough to test the hypothesis at a significance level of 0.05 and power of 0.85. Kolmogorov–Smirnov test was used to evaluate the normality of the data distribution. Kruskal–Wallis, chi-square, and post hoc Tukey tests were used for statistical analysis. *P* values < 0.05 was accepted as statistically significant for Kolmogorov–Smirnov and chi-square tests and less than 0.0125 for post hoc Tukey test. Correlations between CD values and the central corneal thickness and intraocular pressure values were evaluated using Spearman correlation analyzes in the study groups separately.

## Results

The demographic characteristics are summarized in Table 1. The mean age of seven male and six female JOAG cases was 13.54 ± 1.76, the mean age of seven male and five female pseudophakic glaucoma cases was 12.83 ± 1.8, the mean age of five male and eight female aphakic glaucoma cases was 13.46 ± 1.61, and the mean age of eight male and seven female control subjects was 12.13 ± 1.81 years (*p* = 0.057, *p* = 0.76 age and sex, respectively). Time since glaucoma diagnosis was 25.9 ± 12.1 months in JOAG, 28.5 ± 11.3 months in pseudophakic cases, 30.3 ± 11.1 months in aphakic cases.

**Table 1:** The demographics of the cases

Group	JOAG *n*: 13	Pseudophakic *n*: 12	Aphakic *n*: 13	Control *n*: 15	*P* value
Female *n* (%) Male *n* (%)	6 (46.2%) 7 (53.8%)	5 (41.6%) 7 (58.4%)	8 (61.5%) 5 (38.5%)	7 (46.6%) 8 (53.4%)	*p* = 0.76*
Mean age (years ± SD)	13.54 ± 1.76	12.83 ± 1.8	13.46 ± 1.61	12.13 ± 1.81	*p* = 0.057**
Time since glaucoma diagnosis (months ± SD)	25.9 ± 11.1	28.5 ± 11.3	30.3 ± 11.1		

*JOAG* juvenile open-angle glaucoma, *n* number, *SD* standard deviation

*Kruskal–Wallis test

**Chi-square test

The mean IOP values were 15.75 ± 1.6 mmHg in eyes with JOAG, 14.5 ± 1.8 mmHg in pseudophakic eyes, 21.1 ± 2.6 mmHg in aphakic eyes, and 16.2 ± 2.1 mmHg in control subjects. The mean CCT values were, 528.92 ± 17.1 µm in eyes with JOAG, 549.17 ± 15.1 µm in pseudophakic eyes, 612.7 ± 37.8 µm in aphakic eyes and 524.4 ± 18.79 µm in control subjects. The mean IOP values and CCT values were significantly higher in aphakic glaucoma cases than the other groups (*p* = 0.001) (Table 2). Glaucoma was under control in all glaucoma cases with prostaglandin or β-blocker monotherapy or fixed combinations without any clinical, structural, and functional progression for at least one year (Table 2).

**Table 2:** The clinical characteristics of the cases

Group	JOAG *n*: 13	Pseudophakic *n*: 12	Aphakic *n*: 13	Control *n*: 15	*P* value	Post Hoc
Mean IOP (mmHg ± SD)	15.75 ± 1.6	14.5 ± 1.8	21.1 ± 2.6	16.2 ± 2.1	*p* = 0.001*	3 > 1 = 2 = 4
Mean CCT (µm ± SD)	528.9 ± 17.1	549.2 ± 15.1	612.7 ± 37.8	524.4 ± 18.79	*p* = 0.001*	3 > 1 = 2 = 4
Medical agent Β-blocker FC PG	3 eyes 4 eyes 6 eyes	2 eyes 2 eyes 8 eyes	4 eyes 9 eyes			

*JOAG* Juvenile open-angle glaucoma, *IOP* intraocular pressure, *CCT* central corneal thickness

*Statistically significant, PG Prostaglandin monotherapy, FC Βeta blocker- carbonic anhydrase inhibitor fixed combination

The CD values of the groups are summarized in Table 3. In aphakic eyes, the mean CD values were higher in all of the anterior, center, and posterior layers of 0–2 mm, 2–6 mm, 6–10 mm, and total zones (Table 3). In pseudophakic eyes, the mean CD values were statistically similar with that of aphakic eyes and higher than that of JOAG and control eyes in all layers of 0–2 mm zone and in anterior layer of 10–12 mm and anterior and total layers of 2–6 mm zones (Table 3).

**Table 3:** The corneal density values of the groups

	JOAG	Pseudophakic	Aphakic	Control	*P* value	Post hoc
Ant 0–2	17.64 ± 1.64	24.49 ± 7.79	27.19 ± 6.13	18.12 ± 1.99	0.0001*	2 = 3 > 1 = 4
Center 0–2	11.20 ± 1.38	15.67 ± 5.69	18.32 ± 3.41	11.56 ± 1.31	0.0001*	2 = 3 > 1 = 4
Post 0–2	9.16 ± 1.10	12.02 ± 4.05	16.37 ± 3.60	9.07 ± 1.17	0.0001*	2 = 3 > 1 = 4
Total 0–2	12.78 ± 1.23	17.78 ± 6.24	20.47 ± 3.72	13.13 ± 2.18	0.0001*	2 = 3 > 1 = 4
Ant 2–6	16.25 ± 1.39	22.41 ± 7.71	24.35 ± 5.56	16.57 ± 2	0.001*	2 = 3 > 1 = 4
Center 2–6	10.37 ± 1.29	13.87 ± 4.92	17.6 ± 4.18	11.32 ± 2.17	0.0001*	3 > 1 = 2 = 4
Post 2–6	8.76 ± 1.66	11.42 ± 4.87	16.25 ± 3.99	9.35 ± 2.04	0.0001*	3 > 1 = 2 = 4
Total 2–6	12.13 ± 2.08	16.66 ± 6.43	19.85 ± 3.6	12.36 ± 2.23	0.0001*	2 = 3 > 1 = 4
Ant 6–10	15.99 ± 2.52	21.11 ± 6.26	28.1 ± 8.6	15.65 ± 2.16	0.001*	3 > 2 > 1 = 4
Center 6–10	11.08 ± 2.17	14.71 ± 7.17	25.28 ± 9.16	11.53 ± 2.65	0.0001*	3 > 1 = 2 = 4
Post 6–10	9.79 ± 1.71	13.03 ± 7.46	23.58 ± 9.21	9.65 ± 2.44	0.0001*	3 > 1 = 2 = 4
Total 6–10	12.25 ± 2.08	16.85 ± 6.96	25.3 ± 8.84	11.67 ± 1.83	0.0001*	3 > 1 = 2 = 4
Ant 10–12	23.17 ± 6.32	25.44 ± 4.92	28.45 ± 6.25	22.49 ± 4.24	0.033*	2 = 3 > 1 = 4
Center 10–12	15.71 ± 2.68	16.1 ± 4.05	27.7 ± 8.54	15.42 ± 2.78	0.0001*	3 > 1 = 2 = 4
Post 10–12	12.18 ± 3.04	14.2 ± 3.98	26.4 ± 9.12	11.3 ± 1.67	0.0001*	3 > 1 = 2 = 4
Total 10–12	18.18 ± 2.36	18.85 ± 4.52	27.15 ± 6.26	16.93 ± 2.77	0.0001*	3 > 1 = 2 = 4
Total- Ant	17.47 ± 2.08	22.87 ± 6.81	26.71 ± 7.66	17.39 ± 2.6	0.0001*	3 > 1 = 2 = 4
Total-Center	11.75 ± 1.16	14.94 ± 5.83	21.09 ± 4.67	11.71 ± 1.4	0.0001*	3 > 1 = 2 = 4
Total- post	9.45 ± 1.18	12.53 ± 5.56	18.95 ± 4.16	9.7 ± 1.87	0.0001*	3 > 1 = 2 = 4
Total-total	13.45 ± 1.38	17.4 ± 6.05	22.19 ± 4.24	13.3 ± 2.04	0.0001*	3 > 2 > 1 = 4

*Ant* anterior, *Post* posterior

*Statistically significant (Kruskal–Wallis test)

The CD values at total corneal thickness in the 0–2 mm annulus demonstrated significant correlations with CCT values in both aphakic and pseudophakic eyes. However, a significant correlation of CD values with IOP was only demonstrated in aphakic eyes (Table 4).

**Table 4:** Correlation of corneal densitometry at total corneal thickness in the 0–2 mm annulus and age, central corneal thickness, and intraocular pressure

Study groups	CD-Age	*P* value	CD-CCT	*P* value	CD-IOP	*P* value
JOAG	0.033	0.91	− 0.320	0.28	− 0.390	0.18
Pseudophakic	− 0.364	0.24	0.690	0.01*	0.177	0.58
Aphakic	0.046	0.88	0.658	0.01*	0.668	0.01*
Control	0.030	0.91	− 0.400	0.14	− 0.510	0.05

*Statistically significant (Spearman regression analyzes)

## Discussion

Increased CD is related with increased corneal backscatter of light and corneal transparency [10–12]. Even in the absence of clinically significant corneal edema, there can be corneal back-scattering of light so measurement of CD might be an indicator for mild, insignificant corneal edema [10–12]. The corneal stroma maintains its clearness through the regular organization of collagen fibrils within lamellar sheets. Both the spacing of the fibrils within the arrangement and the size of the collagen fibrils effects transparency. Disturbance of the corneal collagen matrix surrounding these collagen fibrils also may influence corneal transparency during edema [13].

Here, in this study, pediatric cases with glaucoma were examined. The purpose of the present study was to compare the CD of eyes with glaucoma following childhood cataract surgery and JOAG and compare the results of them with normal subjects. We found significantly higher CD values in most of the layers and zones in aphakic cases than the other groups. In pseudophakic eyes, the mean CD values were similar with that of aphakic eyes and higher than that of JOAG and control eyes in all layers of 0–2 mm zone and in anterior layers of 10–12 mm and 2–6 mm zones (Table 3).

Increased CD was reported to be related with increased age according to a previous study [10]. The authors investigated healthy cases between 6 and 76 years-old and observed a significant positive correlation between age and increased CD. But no significant correlations were observed between CCT, corneal volume, corneal power, refractive status, and CD values [10]. However, another study could not find any correlations between increased age and CD [14]. In the present study, only pediatric glaucoma cases under 16 years of age were included and the CD values demonstrated no significant correlations with age in all study groups. On the other hand, the CD values demonstrated significant correlations with CCT values in both aphakic and pseudophakic eyes. The CD values also demonstrated significant correlations with IOP values in aphakic eyes. It might be considered that corneal thickness influences the CD measurements in aphakic and pseudophakic glaucoma eyes. Corneal densitometry parameters might be useful for patient follow-up, given the importance of these properties in corneal transparency and their influence on IOP and CCT measurements. CD parameters may have an important effect on interpreting intraocular pressures. The results of this study also support a possible role of CD as a complementary test in the diagnosis and prognosis of glaucoma. However, the relationship between corneal densitometry and IOP and the potential confounding effect of corneal densitometry on the relationship between IOP and corneal thickness should be studied in larger samples. Moreover, a recent study also reported that CD has the potential to serve as a biomarker for early glaucoma risk assessment [15].

Glaucoma may cause some morphological and densitometric corneal changes. Molero-Senosiain et al. [16] compared corneal topography, anterior segment parameters, and CD measurements in patients with POAG and healthy subjects. In topographic and anterior segment analysis, they found significantly higher central anterior elevation, posterior elevation apex, and anterior chamber angle in eyes with POAG. They also found higher CD values in all depth layers in eyes with POAG than the control eyes and positive correlation between age and CD were also demonstrated in the aforementioned study [16]. They showed that CD had a good diagnostic performance for glaucoma. In the present study, we did not find any differences in CD values of JOAG and control subjects different from a previous study [16]. This result may be caused by age-related differences of juvenile and older cases.

The major types of primary childhood glaucoma are congenital, infantile, and juvenile glaucoma, and childhood glaucoma can be more serious than adult glaucoma, more challenging to diagnose and treat [1–4]. Morales-Fernández et al. included primary congenital glaucoma cases to their study and investigated CD, topographic measurements, and biomechanical properties [17]. They observed higher overall CD (especially in anterior layers), lower corneal biomechanical properties, higher mean posterior, central, and anterior elevation and mean keratometry values in glaucoma cases than control subjects. They stated that CD and visual acuity demonstrated a negative significant correlation and measurement of CD might be used as an indirect parameter of BCVA [17]. There were no significant correlations between visual acuity and CD in the present study (*p* > 0.05). Probably, the lack of significance can be attributed to the small sample size and possible unreliable visual acuity levels in pediatric population as a limitation of this study. In the present study, we did not include cases with primary congenital glaucoma and infantile glaucoma because we excluded the severe glaucoma cases. The main reason for this exclusion was the probable negative effect on corneal health of the severity of the disease and previous glaucoma surgeries.

Glaucoma is the most common lifelong complication of pediatric cataract surgery and its incidence is reported to be 15–45% [8, 9]. Almost in one third of the aphakic and pseudophakic glaucoma cases, glaucoma is diagnosed within the first year after cataract surgery but it may occur even after years or decades [8, 9]. Except glaucoma, cataract surgery itself or aphakia-pseudophakia may affect corneal morphology and also CD in these cases. Aphakic patients without glaucoma should also be evaluated to better understand the relationship between aphakia and CD in further studies.

Ishikawa et al. [18] measured CD values after cataract surgery preoperatively and on days 1, 3, and 7 after the surgery. They observed significant increase in CD values on the first day and decrease to preoperative values at the first week. They stated that cataract surgery did not have long-term effect on CD values and the measurement of CD might be a diagnostic tool for undetectable corneal edema by slit-lamp examination [18].

In the present study, our hypothesis was that glaucoma, aphakia, or pseudophakia might have effects on corneal backscatter of light and CD in childhood glaucoma. We aimed to compare the CD values in pediatric glaucoma cases following childhood cataract surgery, JOAG and control subjects. Particularly, aphakia in childhood may affect corneal morphology and topography. Central corneal thickness (CCT) was shown to increase after cataract surgery in childhood and mostly in aphakic eyes [19]. Faramarzi et al. [20] investigated the changes in CCT and corneal biomechanics in eyes with aphakia, primary and secondary pseudophakia following congenital cataract surgery in their study. They showed higher CCT and lower corneal hysteresis in aphakia and secondary pseudophakia groups and stated that primary intraocular lens implantation was the main factor for the preservation of corneal structure and function of the anterior chamber angle [20]. In the present study, all pseudophakic cases had primary IOL implantation. We excluded cases with secondary IOL implantation and clinically complicated cases with microcornea or nystagmus. We also excluded advanced glaucoma cases because of the probable effects of previous glaucoma surgery. We found significantly higher CCT in aphakic glaucoma cases than the other groups like a previous study [18].

A previous study investigated the long-term effects of unilateral pediatric cataract surgery on corneal endothelium and found significantly lower hexagonal cell percentage in aphakic eyes than pseudophakic and un-operated eyes [21]. According to another study, inverse and moderate correlations were found between CD and hexagonal cell percentage [12]. In the present study, probable adverse effects of aphakia on corneal endothelium might have caused the higher CD values in all zones and layers. However, specular microscopy and investigation of the correlations between corneal endothelial parameters and CD values could not be performed in the present study. This is one of the limitations of the present study.

The second limitation is that the cases involved in the present study were on different kinds of anti-glaucoma drops and the effects of drops on CD values were not investigated. Anti-glaucomatous medications may also affect CD values. A previous study investigated the effect of topical latanoprost on corneal clarity in glaucoma cases [22]. Decrease in CD values was observed in aforementioned study. Further prospective studies with a longer follow-up period are required to clarify the relationship especially between prostaglandin analogs and their effects on the cornea transparency. The prostaglandine use in the JOAG and the pseudophakic groups may be a confounding factor in the present study. The mean age at the time of lensectomy was not noted in the present study. The probable earlier mean age at the time of lensectomy in aphakic glaucoma study group is well known to effect CCT and IOP. Early lensectomy can lead to thicker CCT and higher IOP in aphakic glaucoma group and this might be another confounding factor in the present study. Moreover, we could not assess the visual acuity levels, visual field analysis, RNFL measurement and gonioscopy in all cases because of the lack of cooperation in the pediatric population. Cross-sectional design and small sample size are the other limitations of the present study. However, the significance of the findings of the present study lies in that this is the first study to evaluate the corneal densitometry across different glaucoma types of pediatric population.

As conclusion, we observed significantly higher CD values in all layers and zones in aphakic glaucoma cases than pseudophakic glaucoma, JOAG, and control subjects in childhood. To the best of our knowledge the comparison of CD in these pediatric groups has not been studied previously. The probable effects of childhood cataract surgery especially aphakia might cause corneal backscatter of light and increased CD in all layers in all zones of the cornea by affecting corneal morphology. Increased CD values, especially, in aphakic glaucoma and its correlation with CCT and IOP may be considered as a potential implication for clinical management and stimulates further studies. In order to investigate the possible effects of glaucoma on CD values, further investigations about the effects of different types of glaucoma on CD should be encouraged.
